# Efflux pump-deficient mutants as a platform to search for microbes that produce antibiotics

**DOI:** 10.1111/1751-7915.12295

**Published:** 2015-06-08

**Authors:** Carlos Molina-Santiago, Zulema Udaondo, Abdelali Daddaoua, Amalia Roca, Jesús Martín, Ignacio Pérez-Victoria, Fernando Reyes, Juan-Luis Ramos

**Affiliations:** 1 Department of Environmental Protection, Consejo Superior de Investigaciones CientíficasE-18008, Granada, Spain; 2 Bio-Iliberis R&D, Poligono Juncarilcalle Capileira nº 7, E-18121, Peligros, Granada, Spain; 3 Fundación MEDINA, Centro de Excelencia en Investigación de Medicamentos Innovadores en Andalucía. Avda. del Conocimiento 3, Parque Tecnológico de Ciencias de la SaludE-18100, Armilla, Granada, Spain; 1 Abengoa Research, Campus Palmas AltasSevilla, Spain

## Abstract

*Pseudomonas putida* DOT-T1E-18 is a strain deficient in the major antibiotic efflux pump (TtgABC) that exhibits an overall increased susceptibility to a wide range of drugs when compared with the wild-type strain. We used this strain as a platform to search for microbes able to produce antibiotics that inhibit growth. A collection of 2400 isolates from soil, sediments and water was generated and a drop assay developed to identify, via growth inhibition halos, strains that prevent the growth of DOT-T1E-18 on solid Luria–Bertani plates. In this study, 35 different isolates that produced known and unknown antibiotics were identified. The most potent inhibitor of DOT-T1E-18 growth was an isolate named 250J that, through multi-locus sequence analysis, was identified as a *Pseudomonas* sp. strain. Culture supernatants of 250J contain four different xantholysins that prevent growth of Gram-positive bacteria, Gram-negative and fungi. Two of the xantholysins were produced in higher concentrations and purified. Xantholysin A was effective against *Bacillus*, *Lysinibacillus* and *Rhodococcus* strains, and the effect against these microbes was enhanced when used in combination with other antibiotics such as ampicillin, gentamicin and kanamycin. Xantholysin C was also efficient against Gram-positive bacteria and showed an interesting antimicrobial effect against *Pseudomonas* strains, and a synergistic inhibitory effect with ampicillin, chloramphenicol and gentamicin.

## Introduction

The increasing antibiotic resistance level of microorganisms and the emergence of multi-drug-resistant bacteria due to the accumulation of several resistance genes have evoked a critical situation for the treatment of immunosuppressed patients and elderly people who are infected by pathogenic bacteria (Teran *et al*., 2006; Donadio *et al*., 2009). Different modes of antibiotic resistance have been described in bacteria, for instance; acquired resistance, linked to mutations in chromosomal genes or the acquisition of new genes by horizontal transfer (D'Costa *et al*., 2011; Bernal *et al*., 2013); adaptive resistance, which involves a temporary increase in the ability of bacterial cells to survive the action of an antibiotic, mainly as the result of alterations in gene and/or protein expression triggered by environmental conditions (Poole, 2012); and intrinsic resistance, which is not related to antibiotic selection but to the specific characteristics of the bacteria, due to the presence of outer membrane lipopolysaccharides that prevent antibiotic entry and the presence of efflux pumps that extrude antibiotics out of the cells (Lomovskaya and Watkins, 2001,2001; Bernal *et al*., 2013; Li *et al*., 2015).

These variety of resistance mechanisms and the fast evolution of multi-drug pathogen bacteria have made that one of the current challenges for antibiotic therapy is the identification and development of novel and more effective antibiotics to combat them, particularly those resistant to last-line antibiotic agents (Gwynn *et al*., 2010). The main classes of antibiotic drugs commonly used today were discovered in the second half of last century, and their targets are proteins involved in cell-wall biosynthesis (beta-lactams, glycopeptides), cell membrane structures (daptomycin, colistin), type II topoisomerases (fluoroquinolones), ribosome functions (macrolides, aminoglycosides, tetracyclines), transcriptional machinery (rifamycins) and folate biosynthesis (sulfonamides and trimethoprim) (Fernandes, 2006; Lange *et al*., 2007). Therefore, new antimicrobial agents with a greater potential to address the deficiencies of existing classes of antibiotics are needed to combat bacterial resistance (Gwynn *et al*., 2010; Laverty *et al*., 2010). In addition to newly discovered antimicrobials, other strategy could be the use of two or more antibiotics alone or in combination therapies to prevent or delay the emergence of resistant strains and to take advantage of antibiotic synergism as a potential approach to treat infectious diseases (Zhao *et al*., 2002; Mitosch and Bollenbach, 2014). Among the strategies to discover new antimicrobial compounds, chemical modification of existing drugs either with the aim to increase their spectrum of activity or to enhance their activity, and target-directed screening methods to identify new natural compounds are often used (Moellering, 2011). Studies performed by other research groups (Hsieh *et al*., 1998) pointed out the possibility to use hypersensitive strains in the discovery of new antimicrobial compounds.

*Pseudomonas putida* strain DOT-T1E is able to thrive in the presence of high concentrations of organic solvents, such as toluene, (Ramos *et al*., 1995) and toxic compounds such as dyes, heavy metals and a broad array of antimicrobial compounds of different families (Ramos *et al*., 1998; Teran *et al*., 2003; Fernandez *et al*., 2012). The extrusion of antimicrobial compounds is mainly achieved through the action of resistance nodulation cell division (RND) efflux pumps, being TtgABC the most important one in antibiotics extrusion (Mosqueda and Ramos, 2000; Rojas *et al*., 2001; Fernandez *et al*., 2012; Molina-Santiago *et al*., 2014). The lack of efflux pump has been shown to lead to enhanced antibiotic susceptibility as in other microbes such as *Escherichia coli*, i.e. mutants deficient in AcrAB (Okusu *et al*., 1996) or *Pseudomonas aeruginosa*, i.e. mutants deficient in MexAB (Li *et al*., 1998; Poole and Srikumar, 2001).

This study describes a non-directed strategy for the discovery of antimicrobial compounds using a *P. putida* DOT-T1E strain deficient in the TtgABC efflux pump as reporter for the identification of antimicrobial producer bacteria. This methodology allowed us to identify highly active antimicrobials, including compounds of the xantholysin family produced by *Pseudomonas* sp. strain 250J which showed antimicrobial activity against Gram-positive bacteria but also against Gram-negative, which is rarely described for lipodepsipeptides produced by Gram-negative strains.

## Results and discussion

### Method for the identification of antimicrobial-producing bacteria using *P. putida* DOT-T1E-18 as a reporter

We reasoned that *P. putida* DOT-T1E efflux pump-deficient strains would be more sensitive to antibiotics than the wild-type strain and that this hypothesis, if confirmed, would add a relevant strain for screening for new drugs due to the availability of a sensitive strain. To this end, we carried out minimum inhibitory concentration (MIC) assays and inhibition zone experiments for nine different antibiotics using the wild-type DOT-T1E strain and, ∇TtgABC (DOT-T1E-18) and ∇TtgGHI (DOT-T1E-PS28) mutant strains. We found that for the complete set of antibiotics used, the minimal concentration necessary to inhibit DOT-T1E-18 growth was significantly lower than for the wild-type strain and the DOT-T1E-PS28 strain (Table 1). Similar results were found when inhibition zone assays were performed, i.e. the halos in response to a given concentration of antibiotics of different families were largest in the case of the DOT-T1E-18 mutant strain (Fig. S1). Therefore, the strain DOT-T1E-18 inactivated in TtgABC efflux pump showed higher sensitivity to different antibiotic classes. We therefore reasoned that the use of this hypersensitive strain could facilitate the detection of compounds produced by the isolates present in our collection of 2400 environmental bacteria (described in *Materials and methods*).

**Table 1:** Minimum inhibitory concentrations (μ*g* ml^−1^) of antibiotics for *P. putida* DOT-T1E, DOT-T1E-18 and DOT-T1E-PS28 strains at 30°C

Antibiotic (μg/ml)	DOT-T1E	DOT-T1E-18	DOT-T1E-PS28
Nalidixic acid	250	7.8	250
Chloramphenicol	300	70	300
Piperacillin	35	17	35
Ampicillin	625	200	625
Amikacin	2	< 1	2
Ticarcillin	187	4	187
Gentamicin	4	1	2
Ceftriaxone	11	4	11
Tetracycline	8	< 1	8

### Identification of environmental isolates that prevent growth of *P. putida* DOT-T1E-18

We performed inhibition zone assays to determine indicative antimicrobial activity of the environmental isolates against DOT-T1E-18. In these assays, we spread the indicator strain on Luria–Bertani (LB) plates, and once dried, we dropped 3 μl of each isolated bacteria on the dried surface of the LB plates. After 24 h incubation at 30°C, plates were inspected for the appearance of inhibition halos. We found that 150 bacteria from the collection produced an inhibition halo, and they were considered positive candidates for antimicrobial production (Fig. S2). To further test the production of antimicrobials, culture supernatants were harvested and tested to confirm growth inhibition in 96 well-plates. For these assays, overnight cultures were centrifuged and filtered, and 65 μl of the filtrate was placed in wells with 25 μl of LB and 10 μl of diluted DOT-T1E-18 reporter strain. After 24 h, growth was measured to determine the antimicrobial effect of the supernatants. We found that 86 of the 150 culture supernatants tested inhibited growth in this test.

To learn about the potential phylogenetic adscription of the above 86 isolates, fragments of the 16S rRNA gene were polymerase chain reaction (PCR)-amplified and sequenced. blast analysis revealed that some of the isolates could be siblings since they shared 100% of the sequences. This screening reduced the number of potential different microbes to 35 different strains. Of these strains, *Bacillus* and *Pseudomonas* species were the most represented with 12 and eight different members respectively; bacteria of the genus *Enterobacter*, *Raoultella*, *Alishewanella, Rhodococcus*, *Aeromonas*, *Vibrio*, *Shewanella, Acinetobacter, Alcaligenes, Cupravidus and Lysinibacillus* were also identified (Table S1). In addition, we also found eight strains whose 16S rRNAs matched sequences of strains deposited in the databases as ‘uncultured’ microbes.

### Antimicrobial compound detection

**Fig 1:**
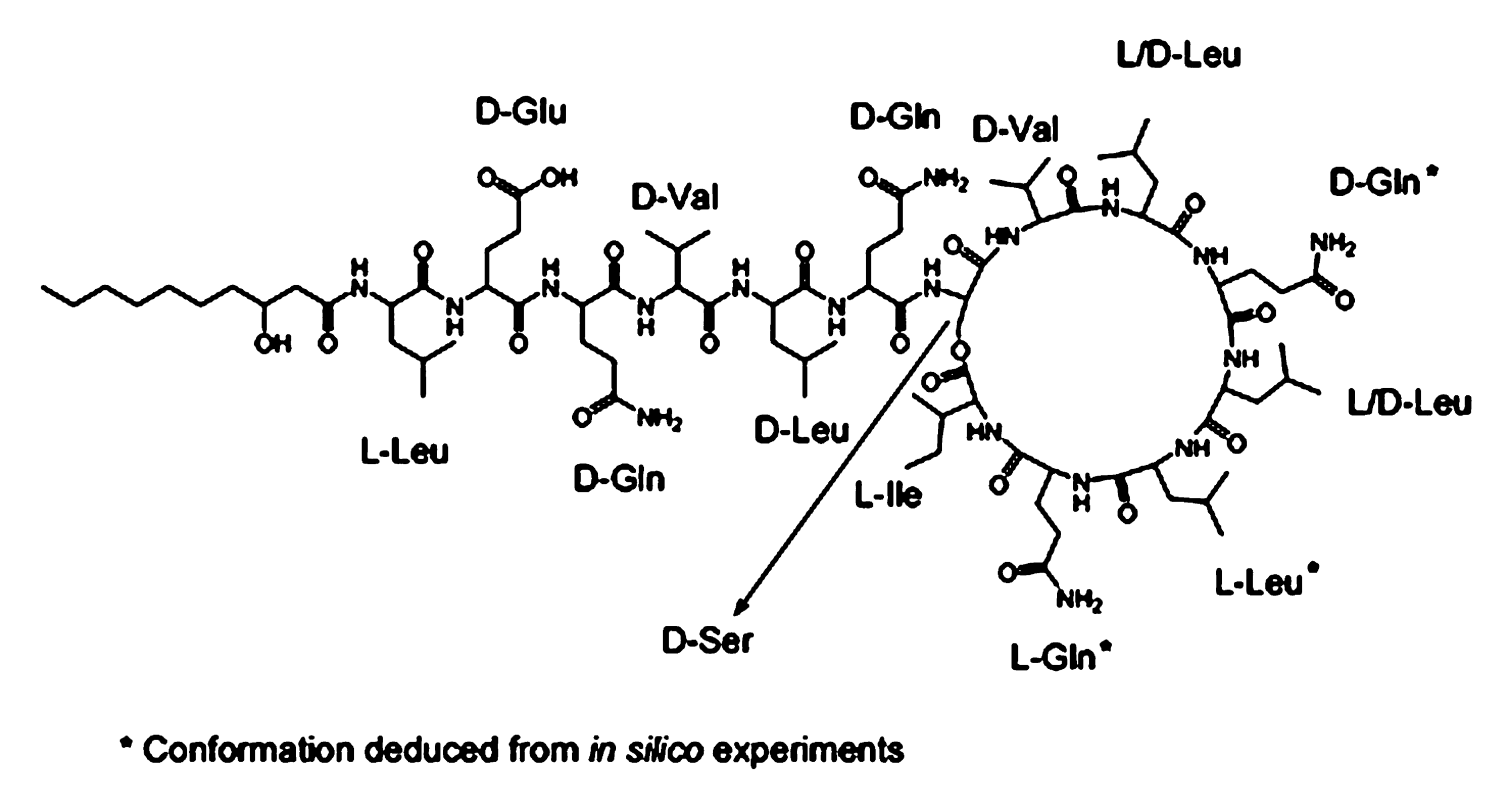
Proposed conformational structure of xantholysin A after Marfey analyses.

Extracts of fermentation broths of the 35 selected strains were analysed by low-resolution mass spectrometry (LR-MS) to detect potential new antimicrobial compounds. The strains were grouped according to their LR-MS profiles, and we found that seven strains were able to produce already described antimicrobials, like *Alishewanella* sp. strain 255W, which produced compounds of the valin-surfactins and plipastatin families (Fig. S3 and Table S2). This information could be used as a proof-of-concept of the screening method. Furthermore, we also identified three strains producing unknown antimicrobial compounds or compounds described in the literature as rarely produced by bacteria. For instance, *Lysinibacillus fusiformis* strain 249MT produced erythrolic acid D and molecules with a known molecular formula which have been described to be produced by fungi, plants and even animals, but not bacteria (Fig. S4 and Table S3). We found that *Vibrio* sp. strain 225TR produced serratomolide C and 4-deoxy thiomarinol H (Fig. S5 and Table S4). Among the isolated strains which produced compounds no identified to the date of the start of our project (December 2012), we found that the largest inhibition halo was produced by the supernatant of *Pseudomonas* sp. strain 250J. In this supernatant, a family of four unknown compounds were detected (Molina-Santiago *et al*., 2015; Table S5). We then decided to study and to analyse the compounds produced by *Pseudomonas* sp. 250J. High-resolution mass-spectrometry (HR-MS) analyses led us to determine that the mass of the compounds were of 1775.08 Da (A), 1761.07 Da (B), 1802.0 Da (C) and 1775.09 Da (D), being compounds A and C the two majoritarian ones (Fig. S6). Therefore, we decided to isolate and to elucidate the conformational structure of these compounds using Nuclear Magnetic Resonance (NMR) and tandem mass spectrometry analyses (Figs S7 and S8). During the elaboration of our analysis, the same compounds were described by Li *et al*., (2013) and named as xantholysins. We not only confirmed the mass of the compounds but also elucidated the conformational structure of the amino acids of xantholysin A (Appendix S1; Fig. 1; Figs S9–S14) using Marfey methodology and high performance liquid chromatography-UV-mass spectrometry (HPLC-UV-MS).

### Antimicrobial activity of xantholysins and synergistic effects with other antibiotics

To assess the antimicrobial activity of the supernatant of *Pseudomonas* sp. strain 250J containing xantholysin, we evaluated its potential against Gram-negative and Gram-positive bacteria, such as different *Pseudomonas* species, *Bacillus subtilis*, *Rhodococcus erythrophyla*, *E. coli* K12 and *Lysinibacillus fusiformis*. For this, culture supernatant of 250J strain was used in growth inhibition assays (Table 2).

**Table 2:** Inhibition growth activity of 250J supernatant and pure xantholysin A and C against Gram-positive and Gram-negative bacteria

	250J Supernatant	Xantholysin A	Xantholysin C
Strains	Growth inhibition (%)	MIC (μg/ml)	
*P. putida* DOT-T1E	79.36	500	62.5
*P. putida* DOT-T1E-18	83.11	500	62.5
*P. putida* KT2440	75.39	500	125
*P. aeruginosa* PAO 1	68.49	> 1000	62.5–125
*P. mendocina*	80.11	500	125
*P. nitroreducens*	85.44	250	125
*E. coli* K12	76.53	> 1000	> 500
*Bacillus* sp.	95.62	50	250
*Lysinibacillus fusiformis*	100	25	31.25
*Rhodococcus erythrophyla*	100	15.7	7.5

Purified xantholysin A inhibited growth of Gram-positive bacteria *Bacillus* sp., *L. fusiformis* and *R. erythrophyla* (MIC of 15–50 μg ml^−1^). Xantholysin A also inhibited growth of Gram-negative strains but higher concentrations were needed, i.e. *Pseudomonas* strains (MIC of 250–500 μg ml^−1^), *E. coli* and *P. aeruginosa* (> 1000 μg/ml) (Table 3). In contrast, when we used purified xantholysin C, we observed growth inhibition in all *Pseudomonas* tested (Table 2) in ranges between 62.5 μg/ml and 125 μg/ml, a fourfold stronger effect than xantholysin A. Furthermore, different to the observed with xantholysin A, xantholysin C is able to inhibit growth of *P. aeruginosa* PAO1, a pathogen strain. However, it is interesting to remark that, although the detection and isolation of a new xantholysin producer was performed using DOT-T1E-18, xantholysin compounds showed similar antimicrobial activity against wild-type and mutant strains. The different effect of molecules of the same family could be indicating that 250J presents an armory of compounds to combat against other microorganisms present in the same ecological niche, or its capacity to colonize other environmental places.

**Table 3:** Synergy of (i) xantholysin A and (ii) xantholysin C with ampicillin, chloramphenicol, kanamycin and gentamicin against Gram-positive and Gram-negative bacteria. Legend: A, additive; S, synergy; I, indifference; nt, not treated

Xantholysin A				
	Ap	Cm	Km	Gm
*P. putida* DOT-T1E	A (0.98)	S (0.49)	A (0.73)	I
*P. putida* KT2440	I	I	I	I
*P. aeruginosa* PAO1	I	I	I	S (0.5)
*Bacillus* sp.	S (0.39)	Nt	I	S (0.5)
*Rhodococcus erythrophyla*	S (0.4)	A (0.75)	S (0.45)	I
*E. coli* K12	A (1)	nt	A (1)	A (1)

Xantholysin C				
	Ap	Cm	Km	Gm
*P. putida* DOT-T1E	I	S (0.44)	A (0.96)	I
*P. putida* KT2440	I	I	A (0.98)	A (0.98)
*P. aeruginosa* PAO1	I	I	I	I
*Bacillus* sp.	S (0.48)	nt	A (0.94)	S (0.49)
*Rhodococcus erythrophyla*	A (0.9)	A (0.9)	I	A (0.9)
*E. coli* K12	A (1)	nt	A (1)	S (0.5)

To analyse a hypothetical role of xantholysins as adjuvant of antibiotics against different strains, checkerboard assays were performed to study synergism of xantholysins A and C with classical antimicrobial compounds like ampicillin, gentamicin, chloramphenicol and kanamycin (Table 3). According to the results shown in Table 3, xantholysin A presented a synergistic effect (FIC ≤ 0.5) when it was used along with ampicillin (*Bacillus sp*. and *Rhodococcus erythrophyla*), gentamicin (*Bacillus sp.*), chloramphenicol (*P. putida* DOT-T1E) and kanamycin (*R. erythrophyla*). As shown in Table 3, additive effects were found with xantholysin A in combination with some antibiotics, while in other cases, synergistic effects were not found. In the case of xantholysin C, we only observed synergistic effects when it was supplied with chloramphenicol against DOT-T1E, with ampicillin and gentamicin against *Bacillus sp*., and with gentamicin against *E. coli* K12 (Table 3).

## Concluding remarks

The emergence of multi-resistant bacteria and the lack of new antibiotics to combat them has become a serious risk for humanity. One class of compounds that, during the recent decades, have attracted an increasing amount of attention due to their promising role as therapeutics or drug leads are short antimicrobial peptides that usually exhibit rapid and efficient antimicrobial toxicity against a wide range of pathogens, mainly Gram-positive strains (Ganz and Lehrer, 1999; Hadley and Hancock, 2010; Hughes and Fenical, 2010; Laverty *et al*., 2010). In this study, we provide a platform for the detection of antimicrobial compounds based on a *P. putida* DOT-T1E strain deficient in the TtgABC efflux pump, the main element required for antibiotic extrusion. The mutant strain is more sensitive to antibiotics, making it a more responsive method for the detection of antimicrobials.

Out of the 2400 isolates previously obtained from different environmental niches, we identified 35 antimicrobial producing strains that belonged to different genera; with a predominance of *Bacillus* and *Pseudomonas* strains. High-performance liquid chromatography–mass spectrometry analyses of the culture supernatants from the strains led us to identify different known and unknown antimicrobial compounds, confirming that the platform fulfils the proof-of-concept for detection of microbes that produce antibiotics.

*Pseudomonas* sp. strain 250J was the most promising one, due to its ability to produce a family of, in the discovery moment, four unknown compounds further described as xantholysins. Purified xantholysin A and C were used to elucidate their structures and their L- and D-amino acid configurations. Analysis of their antimicrobial activities showed that both compounds had similar antimicrobial patterns against Gram-positive bacteria. However, xantholysin C also exhibited an interesting activity against Gram-negative bacteria, including *P. aeruginosa*. Further analysis also showed a synergistic effect of both compounds in combination with classical antibiotics against Gram-positive and Gram-negative strains. Taken together, these results support an interesting method for the detection of antimicrobial compounds with higher sensitivity. Further studies are needed to determine the potential use of these compounds in clinical assays.

## Material and methods

### Strains and general growth conditions

The bacterial strains used in this study are shown in Table 4. Bacterial cells were grown in liquid LB medium at 30°C in an orbital shaker at 200 r.p.m. (Kühner). When necessary, the appropriate antibiotics were added to reach the following final concentrations: 50 μg ml^−1^ kanamycin and 20 μg ml^−1^ rifampicin. The concentration of other antibiotics used in this study is indicated in the text. For production of xantholysins, KIDO medium (glycerol 2% [v/v]; sodium L-glutamate monohydrate 1% [w/v]; yeast extract 0.25% [w/v] adjusted to pH 7) was used and cultures were grown at 18°C for 3 days (Pascual *et al*., 2014).

**Table 4:** Strains used in this study

Strain	Relevant characteristics	Source of reference
*Pseudomonas* strains		
*P. putida* DOT-T1E	Rif^r^	Ramos *et al*., 1998
*P. putida* DOT-T1E-18	Rif^r^, Km^r^, *ttgB*::‘*phoA*-Km	Ramos *et al*., 1998
*P. putida* DOT-T1E-PS28	Rif^r^, Sm^r^, *ttgH*ΩSm	Rojas *et al*., 2001
*P. aeruginosa* PAO1	Wild type, prototroph, Ap^r^	Stover *et al*., 2000
*Pseudomonas sp*. strain 250J	Xantholysin producer isolated from the garden of Estación Experimental del Zaidín (Granada, Spain)	This study
*Pseudomonas mendocina*	Isolated from olive soil (Jaén, Spain)	This study
*Pseudomonas nitroreducens*	Isolated from Tinto River (Huelva, Spain)	This study
*Bacillus* strains		
*Bacillus sp.*	Isolated from Tinto River (Huelva, Spain)	This study
*Bacillus subtilis*	Isolated from Muelle del Tinto (Huelva, Spain)	This study
Other strains		
*Lysinibacillus fusiformis*	Isolated from Muelle del Tinto (Huelva, Spain)	This study
*Rhodococcus erytrophyla*	Isolated from Tinto River (Huelva, Spain)	This study
*E. coli* K12	Wild-type	Kar *et al*., 2005

### Sample collection

Samples were collected from soils, wastewater, river waters and river sediments. A loam soil sample was collected from olive fields (Jaén, Spain) in September 2010 [37° 46′ 57.93′ N, −3° 48′ 8.95″ W] at 574 m. A second soil sample was collected from the gardens of the Estación Experimental del Zaidín (Granada, Spain) in November 2010, [+37°9′56.50″N, −3°35′31.13″O] at 678 m. In both cases, 300–500 g of the top 20 cm of soil was collected, placed in sterile 50 ml tubes and kept at −20°C until used.

A five hundred millilitre wastewater sample, which was kept at 4°C until it was used, was collected from a treatment plant (Cartagena, Spain) in November 2010 [+37°36′00″N, −0°59′00″O]. This sample was used to search for microbes that have been exposed to a wide range of domestic waste-derived chemicals, including emerging pollutants (pharmaceuticals and household cleaning products, etc.).

Yellow-red clay samples were collected from the Tinto River (Huelva, Spain) in September 2012 [+37° 18′ 41.79″ N, −6° 49′ 20.62″ W]. The river represents an extreme environment [pH 3–4, heavy metal concentrations of 20 μg ml^−1^ Zn, 180 μg ml^−1^ Fe, 19 μg ml^−1^Cu and 355 μg ml^−1^ S (Garcia-Moyano *et al*., 2012) and high salt concentration due to the proximity to Atlantic Ocean combined with tidal pulls].

Sand samples were also taken along the Odiel River (Huelva, Spain) in September 2012 from [+37° 15′ 2.74″ N, −6° 57′ 27.28″ W] to [+37° 12′ 58.99″ N, −6° 56′ 46.81″ W]. The estuaries of the Odiel River (Huelva, Spain) are characterized by tidal pulls and a high salt concentration due to their proximity to the Atlantic Ocean. Three hundred millilitres water samples were taken from the river channel and kept at 4°C until used. Sediments were sampled when the tidal influence in the river was maximal and minimal. In each case, the samples were prepared by collecting 100 g sediments from each location at sample points that were least 10 m apart.

### Isolation media

The following isolation media with adjusted pH between 7.0–7.5 were used: M9 minimal medium with different carbon sources (syringic acid, vanillic acid, *o*-anisic acid, *p*-anisic acid, ferulic acid, veratric acid, *p*-cumaric acid, 2-hydroxyphenylacetic acid, 2,4-dihydroxybenzoic acid, phloroglucinol, benzoic acid) (Abril *et al*., 1989); IM1 [humic acid agar with sea water, including humic acid (1 g), K_2_HPO_4_ (0.5 g), FeSO_4_ x 7H_2_O (1 mg), agar (20 g), vitamin B solution (1 ml), natural sea water (0.5 L) and distilled water (0.5  L)] (Zotchev, 2012); IM2 [glycerol (0.5 g), starch (0.5 g), sodium propionate (0.5 g), KNO_3_ (0.1 g), asparagine (0.1 g), casein (0.3 g), K_2_HPO_4_ (0.5 g), FeSO_4_ x 7H_2_O (1 mg), agar (20 g), vitamin B solution (1 ml), natural sea water (0.5  L) and distilled water (0.5 L)] (Zotchev, 2012); IM3 [chitin agar with sea water, chitin (Sigma), K_2_HPO_4_ (0.5 g), FeSO_4_ x 7H_2_O (1 mg), agar (20 g), vitamin B solution (1 ml), natural sea water (0.7 L) and distilled water (0.3 L)] (Bredholt *et al*., 2008); IM4 [malt extract (1 g), glycerol (1 g), glucose (1 g), peptone (1 g), yeast extract (1 g), agar (20 g), natural sea water (0.5 L) and distilled water (0.5 L)] (Bredholt *et al*., 2008); IM5-soil agar [filtered soil extract (1 L), agar (20 g), pH 7.0 (soil extract was prepared by mixing 200 g soil in 1 L water, followed by boiling the mixture for 30 min), and 1 ml of vitamin complex solution, comprising calcium pantothenate (10 mg), nicotinic acid (10 mg), thiamin chloride (1 mg), biotin (1 mg) and tap water (20 ml); IM6 [IM5-soil agar with 3% (w/v) sea salt added]; IM7 [modified organic agar 2 Gause, comprising peptone (5 g), tryptone (3 g), glucose (10 g), NaCl (5 g), tap water (1L) (Engelhardt *et al*., 2010), IM8 modified organic agar 2 Gause with 3% (w/v) sea salt added]; IM9-mineral [agar 1 Gause, comprising starch-soluble (20 g), K_2_HPO_4_ (0.5 g), MgSO_4_ (0.5g), KNO_3_ (1.0 g), NaCl, (0.5 g), FeSO4 x 7H_2_O (0.01 g) and tap water (1 L)]; IM10 [modified organic agar 2 Gause supplemented with tobramycin (10 μg ml^−1^)] (Terekhova *et al*., 1991); IM11-½ ISP2 [malt extract (5 g), yeast extract (2 g), glucose (2 g), natural sea water (0.5 L) and distilled water (0.5 L) (Hakvag *et al*., 2008); modified IM12-Kusters streptomycete isolation medium [glycerol (10 g), casein (0.3 g), KNO_3_ (2 g), FeSO_4_ x 7H_2_O (0.25 mg), H_2_SO_4_ (0.5 mg), natural sea water (0.5 L) and distilled water (0.5 L)]; IM13-medium A [9K mineral medium supplemented with 1% (w/v) glucose and 1% (w/v) yeast extract]; IM14-medium I [9K medium supplemented with 0.1% (w/v) bactotryptone, 1% (w/v) malt extract, 1% (w/v) glucose, 0.5% (w/v) yeast extract and 0.5% (w/v) sucrose] (Silverman and Lundgren, 1959); IM15-medium J [9K medium supplemented with 0.1% (w/v) casamino acids, 0.1% (w/v) bactopeptone, 0.5% (w/v) yeast extract and 0.5% (w/v) sucrose]; IM16-medium F [1 mM KH_2_PO_4_, 1 mM MgCl_2_, 1.5 mM (NH_4_)_2_SO_4_, 0.5% (w/v) glucose, 0.05% (w/v) malt extract, 0.5% (v/v) trace metals] (Silverman and Lundgren, 1959).

Inoculated media were incubated at 30°C for 24 h, 48 h, 72 h, 1 week and 2 weeks with agitation (200 rpm). At the indicated times aliquots were collected and plated in the same isolation medium that were used for enrichment, but were supplemented with agar (2% w/v). Different colonies were selected according to their apparent phenotype, such as colour, roughness, size and morphology in order to keep as many different bacteria as possible and enabled creation of a collection of around 2400 microorganisms.

### Phylogenetic analysis

Genomic DNA was used as a template for PCR amplification. For 16S ribosomal RNA (rRNA) gene amplification, primers 27f (5′-AGAGTTTGATCMTGGCTCAG-3′) and 1492r (5′- TACGGYTACCTTGTTACGACTT-3′) were used (Weisburg *et al*., 1991). Polymerase chain reaction amplifications were carried out under the following conditions: initial denaturation at 94°C for 5 min, followed by 30 cycles of 94°C for 30 s, 46°C for 30 s and 72°C for 2 min, with a final extension at 72°C for 7 min. In all cases, the reaction mixture (50 μl) contained deoxyribonucleotide triphosphate (0.2 mM each), 1 x reaction buffer (20 mM Tris pH 8.4, KCl 50 mM), MgCl_2_ (1.7 mM), primers (0.1 μM each) and Taq DNA polymerase (1.25 units). Polymerase chain reaction products were analysed by agarose gel electrophoresis, which were purified using Qiaquick gel extraction kit (Qiagen) and were sequenced directly using primers 27f and 1492r. The partial 16S rRNA gene sequences were analysed using blast (Altschul *et al*., 1997) and named according to their closest neighbours.

### Antimicrobial producer identification

*Pseudomonas putida* DOT-T1E-18 (TtgABC mutant) reporter strain was grown aerobically at 30°C. Antimicrobial activity of the isolated bacteria against the reporter strain was assessed with a colony overlay assay. The indicator bacteria was spread on LB plates and allowed to dry; subsequently, isolated bacterial colonies from an overnight pre-culture were loaded. Plates were incubated for 24 h, and inhibition zones around the spots were scored as positive. In the case of a positive result, liquid antimicrobial activity screening assays were carried out in 96-well plates. These assays were carried out in microdilution plates in which 65 μl of filtrated supernatant was mixed with 25 μl LB and 10 μl of the reporter bacterium that allowed to reach an OD_660nm_ of 0.05. These plates were incubated at 30°C with agitation (200 rpm) for 24 h and growth was assayed. Inhibition was scored as positive if the turbidity was 10-fold lower than in the control without filtrate supernatant. Experiments were repeated in triplicate.

### Checkerboard analysis

Standard powder forms of ampicillin, chloramphenicol, gentamicin and kanamycin were stored at 2°C to 8°C until use. Immediately prior to testing, the stock solutions and serial twofold dilutions of each drug were prepared at concentrations that were at least double the MIC, according to National Committee for Clinical Laboratory Standards (NCCLS) recommendations. A total of 100 μl of LB broth inoculated with the bacterial inoculum at a 0.5 McFarland turbidity was distributed into each well of the microdilution plates. The first drug of the combination was serially diluted along the ordinate, while the second drug was diluted along the abscissa. Plates were incubated at 30°C or 37°C for 24 h at 200 r.p.m.

In accordance with the NCCLS guidelines for broth microdilution, the MIC was defined as the lowest concentration of antibiotic that completely inhibited the growth of the organism as detected by reading turbidity at 600 nm. Synergy is more likely to be expressed according to ΣFICs that were calculated as follows: ΣFIC = FIC A + FIC B, where FIC A is the MIC of drug A in the combination/MIC of drug A alone, and FIC B is the MIC of drug B in the combination/MIC of drug B alone. The combination is considered synergistic when the ΣFIC is ≤ 0.5, additive when the ΣFIC is > 0.5 to ≤ 1, indifferent (no interaction) when ΣFIC is > 1 to ≤ 4 and antagonistic when the ΣFIC is ≥ 4 (Sopirala *et al*., 2010).

### MIC

Minimum inhibitory concentration assays of DOT-T1E, T1E-18 and T1E-PS-28 were performed in liquid LB medium using the twofold serial dilution test according to the Clinical and Laboratory Standards Institute guidelines (2003). The highest concentration of the antibiotics used were: tetracycline (10 000 μg ml^−1^); chloramphenicol (3000 μg ml^−1^); norfloxacin (200 μg ml^−1^); erythromycin (3000 μg ml^−1^); gentamicin (1000 μg ml^−1^), kanamycin (2500 μg ml^−1^) and ampicillin (10 000 μg ml^−1^). In the cases of xantholysin A and C, a concentration of 2000 μg ml^−1^ was used. At least three independent experiments were carried out for each determination, and each experiment was run in triplicate. The minimum inhibitory concentration (MIC) was determined as the lowest concentration of antibiotic that inhibited the growth of the strain by > 90%.

### Disc diffusion antibiotic susceptibility testing

The Kirby–Bauer technique was used (Bauer *et al*., 1966). Briefly, LB agar plates were spread with a suspension of approximately 10^8^ CFU/ml of wild-type *P. putida* DOT-T1E or DOT-T1E-18 mutant strain to produce a lawn. Once the plate surface was dried, antibiotic disks of ofloxacin (5 μg), pefloxacin (5 μg), amoxicillin (25 μg), ticarcillin (75 μg), ampicillin (10 μg), ceftazidime (30 μg), chloramphenicol (30 μg), erythromycin (15 μg) and tetracycline (30 μg) (BioMerieux, Spain) were placed on the surface of plates. After 18–20 h at 30°C, the inhibition zone was measured around each disc.

### Extraction and purification of xantholysin A and xantholysin C

A culture of *Pseudomonas* sp. strain 250J (1 L) grown in 2 L flasks for 3 days at 18°C was extracted by acidifying the fermentation broth with HCl to a pH of 2–3. Subsequently, methyl ethyl ketone (EtCOMe) was added and three 10 min centrifugation steps were carried out. After each centrifugation, the organic phase was transferred to glass tubes. The sample was evaporated on an orbital shaker to concentrate the sample and re-suspended in dimethylsulphoxide. The solution was loaded onto a semi-preparative HPLC column [Zorbax SB-C8, 21.2_250 mm, gradient H_2_O 0.1% (v/v) CF_3_COOH (TFA)/Acetonitrile 0.1% (v/v) TFA from 5% to 100% (v/v) of Acetonitrile/TFA in 45 min, 3.6 ml min^−1^, UV detection). The peak eluting at 31 min was re-purified on the same column with a 68% isocratic Acetonitrile/TFA yielding 16 mg of xantholysin A and 5 mg of xantholysin C.

### Preparation and analysis of Marfey derivatives

Xantholysin A was hydrolysed by heating in HCl (6 N, 300 μL) at 110°C for 16 h (total hydrolysis) and in HCl (0.5 N) at 110°C for 7 h (partial hydrolysis). In the case of partial hydrolysis, fragments of interest were purified by HPLC using a gradient of 30–80% (v/v) of acetonitrile in water. After cooling, the solution was evaporated to dryness and re-dissolved in H_2_O (50 μL). NaHCO_3_ (1 M, 20 μL) was added to the peptide acid hydrolysate solution (or to 50 μL of a 50 mM solution of the respective amino acid standard), and then a 1% (w/v) solution (100 μL) of FDVA (Nα-(2,4-dinitro-5-fluorophenyl)-L-valinamide, a variant of Marfey's reagent) in acetone. The mixture was incubated for 1 h at 40°C. The reaction was stopped by addition of HCl (1 N, 20 μL). A 3 μL aliquot of these solutions were analysed by HPLC-UV-MS (Zorbax SB-C8 column, 2.1 × 30 mm, 5 μm, 40°C, 500 μL/min; linear gradient: 0 min 22% B, 33 min 60% B, mobile phase and detection as described above for the general LC-UV-MS analysis). Retention times (min) of the FDVA amino acid derivatives used as standards were as follows: L-Ser (7.6), D-Ser (9.5), L-Glu (8.9), D-Glu (11.6), L-Val (17.1), D-Val (25.1), L-Leu (20.8), D-Leu (29.2), L-Ile (20.8) and D-Ile (29.2).

Retention times (min) of the observed peaks in the HPLC trace of the FDVA-derived hydrolysis products of xantholysin A were as follows: D-Ser (9.7), L-Glu (9.4), D-Glu (11.9), D-Val (25.1), L-Leu (20.8), D-Leu (29.1), L-Ile (20.8) and D-Ile (29.1). Due to the close retention times observed for L-Ile, L-Leu, D-Ile and D-Leu, the presence of L-Ile or D-Ile was confirmed using a second HPLC method with isocratic elution (33% B during 60 min). Under these conditions, retention times of 19.1 and 19.9 min were obtained for L-Ile and L-Leu respectively.

## Conflict of interest

None declared.

## Supporting Information

Fig S1 Inhibition halos produced by different antibiotics against (A) *P. putida* DOT-T1E, (B) *P. putida* DOT-T1E-18 and (C) *P. putida* DOT-T1E-PS28. From left to right and top to bottom: ofloxacin, ciprofloxacin, amoxicillin, ticarcillin, ampicillin, chloramphenicol, ceftazidime, erythromycin and tetracycline.

Fig S2 Inhibition halos produced by different bacteria against the reporter strain DOT-T1E-18.

Fig S3 High-performance liquid chromatography–mass spectrometry profile of 255W culture extracts.

Fig S4 High-performance liquid chromatography–mass spectrometry profile of 249MT culture extracts.

Fig S5  High-performance liquid chromatography–mass spectrometry profile of 225TR culture extracts.

Fig S6 High-performance liquid chromatography–mass spectrometry profile of 250J culture extracts.

Fig S7 Nuclear Magnetic Resonance (NMR) of xantholysin A in MeOD.

Fig S8 Tandem mass spectrometry analysis of xantholysin C. The analyses were performed to confirm the difference in the lipid tail between xantholysin A and xantholysin C, proposed by (Bernal *et al*., 2013).

Fig S9 Marfey analysis of D-Ser, (A) xantholysin A MS, (B) positive ion of D-Ser in xantholysin A and (C) positive ion of D-Ser control.

Fig S10 Marfey analysis of D-Val. (A) xantholysin A MS, (B) positive ion of D-Val in xantholysin A and (C) positive ion of D-Val control.

Fig S11 Marfey analysis of L- and D-Glu/Gln. (A) xantholysin A MS, (B) positive ion of D- and L-Glu/Gln in xantholysin A, (C) positive ion of D-Glu/Gln control and (D) positive ion of L-Glu/Gln control.

Fig S12 Marfey analysis of D- and L-Ile/Leu. (A) xantholysin A MS, (B) positive ion of D- and L-Leu/Ile in xantholysin A, (C) positive ion of D-Leu control, (D) positive ion of D-Ile control, (E) positive ion of L-Leu control and (F) positive ion of L-Ile control.

Fig S13 Marfey analysis of a fragment of 301 Da from partial hydrolysis. (A) fragment of 301 Da MS, (B) positive ion of L-Leu in fragment of 301 Da, (C) positive ion of L-Leu control.

Fig S14 Marfey analysis of a fragment of 900 Da from partial hydrolysis. (A) Fragment of 900 Da MS, (B) positive ion of D-Leu and L-Leu in fragment of 900 Da, (C) positive ion of D-Leu control, (D) positive ion of L-Leu control, (E) positive ion of D-Glu in fragment of 900 Da, (F) positive ion of D-Glu control.

Table S1 Isolation of species capable of inhibiting DOT-T1E-18 growth.

Table S2 Antimicrobial compounds found in the 255W culture extracts. Positions correspond with the numbers that appear in Fig. S2.

Table S3 Antimicrobial compounds found in the 249MT extract. Positions correspond with the numbers that appear in Fig. S3. NPB, not produced by bacteria.

Table S4 Antimicrobial compounds found in the 225TR extract. Positions correspond with the numbers that appear in Fig. S4. DNP, not described in database of natural products.

Table S5 Antimicrobial compounds found in the 250J extract. Positions correspond with the numbers that appear in Fig. 1.

Appendix S1 Marfey analysis of xantholysin A.
