# Supplementary figures and images for: Efflux pump-deficient mutants as a platform to search for microbes that produce antibiotics
Source: Microb Biotechnol. 2015 Jun 8;8(4):716–25. doi: 10.1111/1751-7915.12295 (PMC4476826; doi:10.1111/1751-7915.12295)

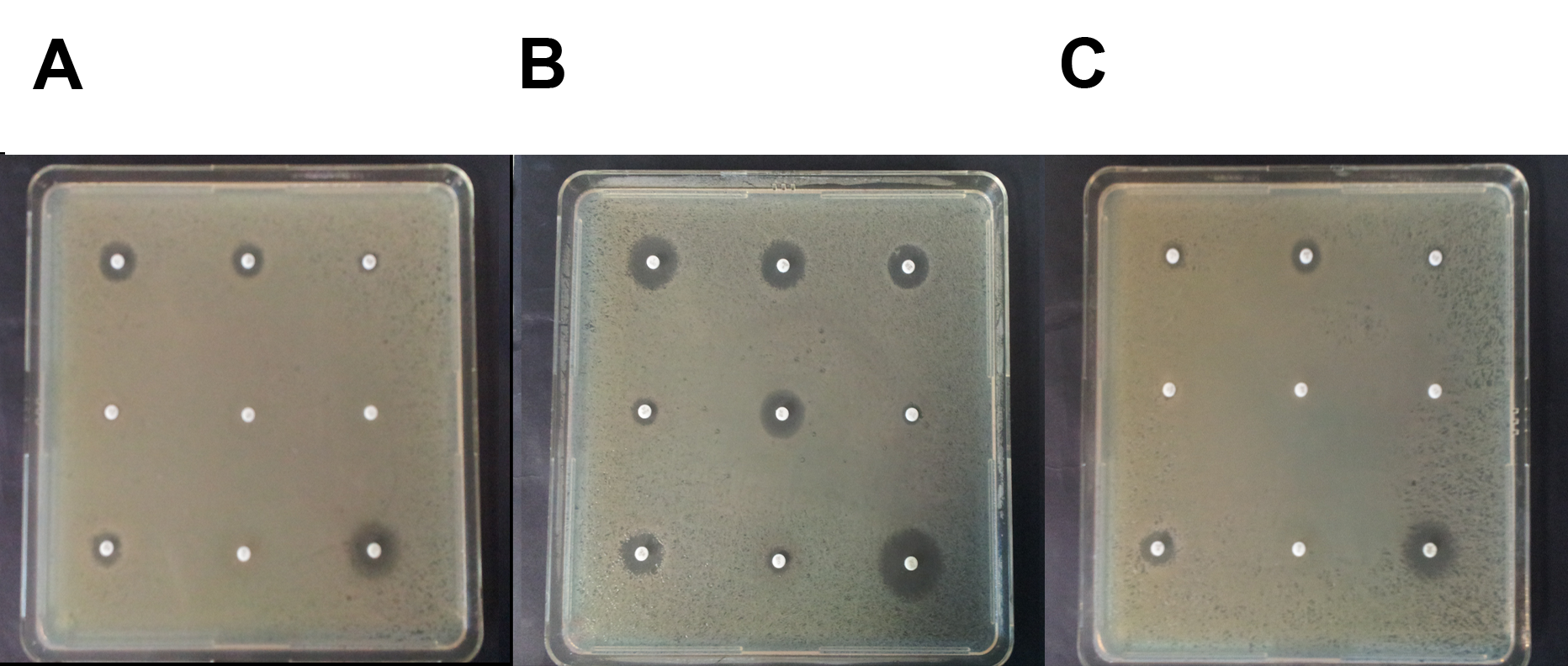

Supplement: Fig S1 — Inhibition halos produced by different antibiotics against (A) P. putida DOT-T1E, (B) P. putida DOT-T1E-18 and (C) P. putida DOT-T1E-PS28. From left to right and top to bottom: ofloxacin, ciprofloxacin, amoxicillin, ticarcillin, ampicillin, chloramphenicol, ceftazidime, erythromycin and tetracycline. [file mbt20008-0716-sd1.tif]

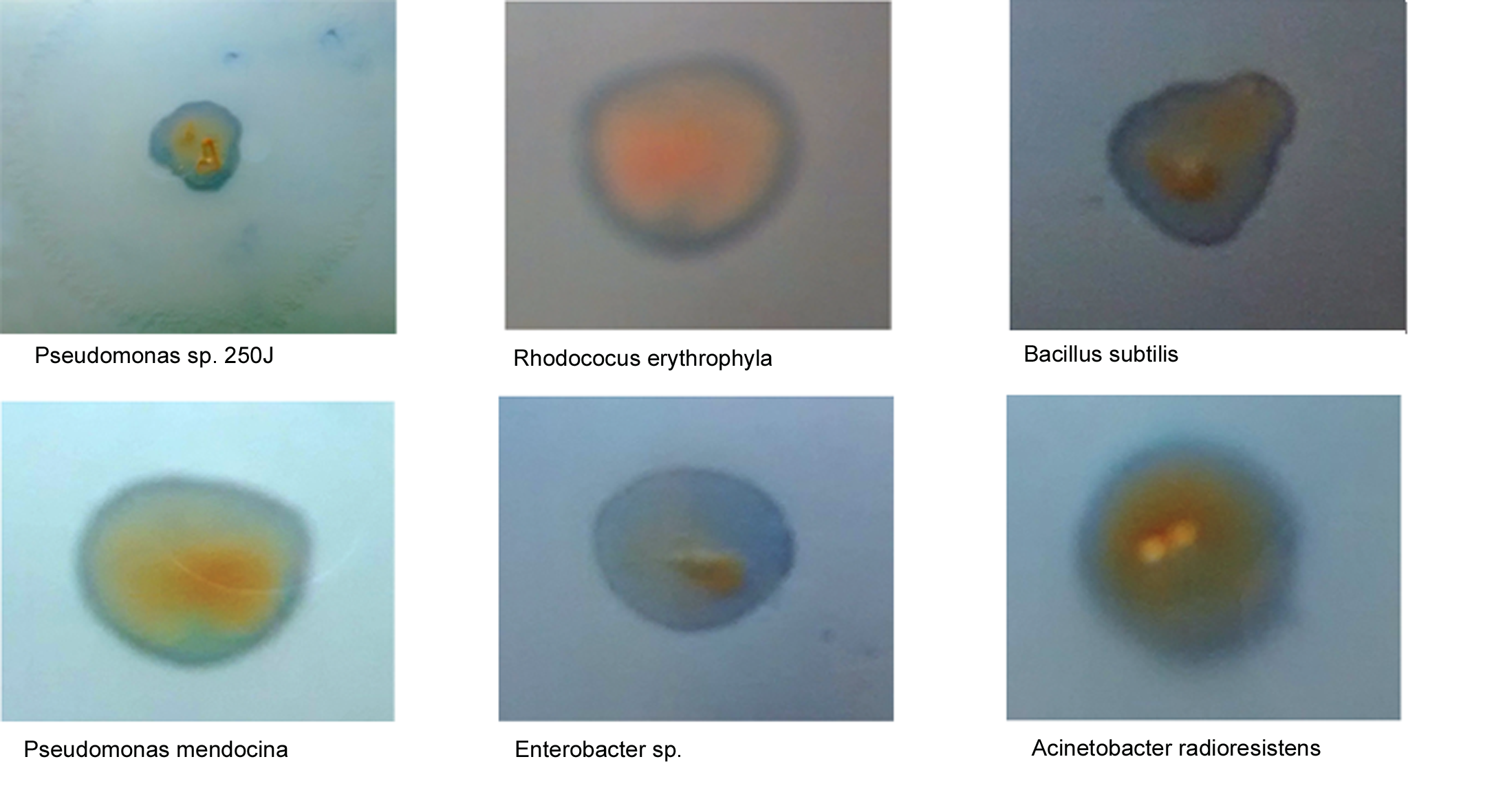

Supplement: Fig S2 — Inhibition halos produced by different bacteria against the reporter strain DOT-T1E-18. [file mbt20008-0716-sd2.tif]

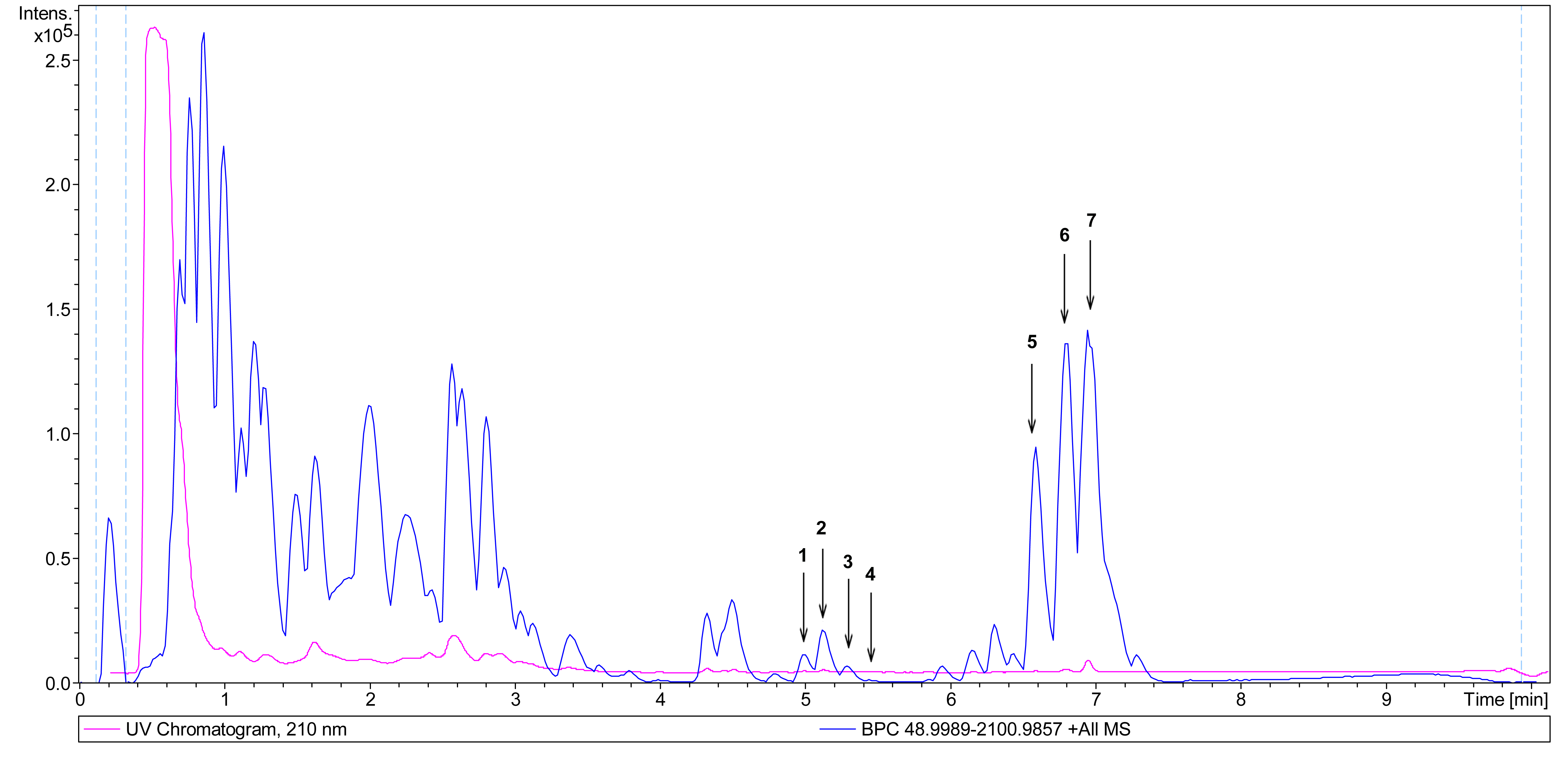

Supplement: Fig S3 — High-performance liquid chromatography–mass spectrometry profile of 255W culture extracts. [file mbt20008-0716-sd3.tiff]

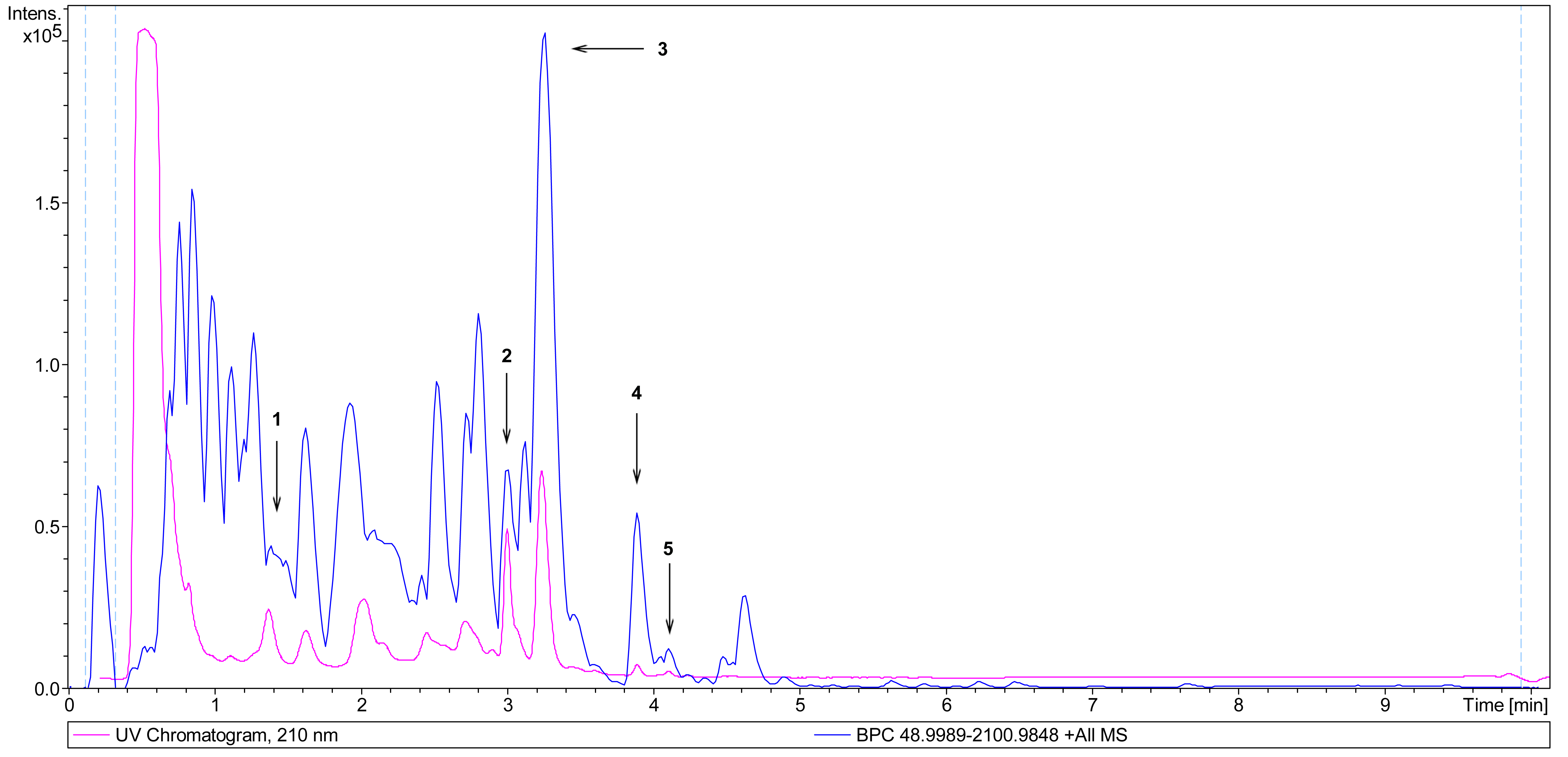

Supplement: Fig S4 — High-performance liquid chromatography–mass spectrometry profile of 249MT culture extracts. [file mbt20008-0716-sd4.tiff]

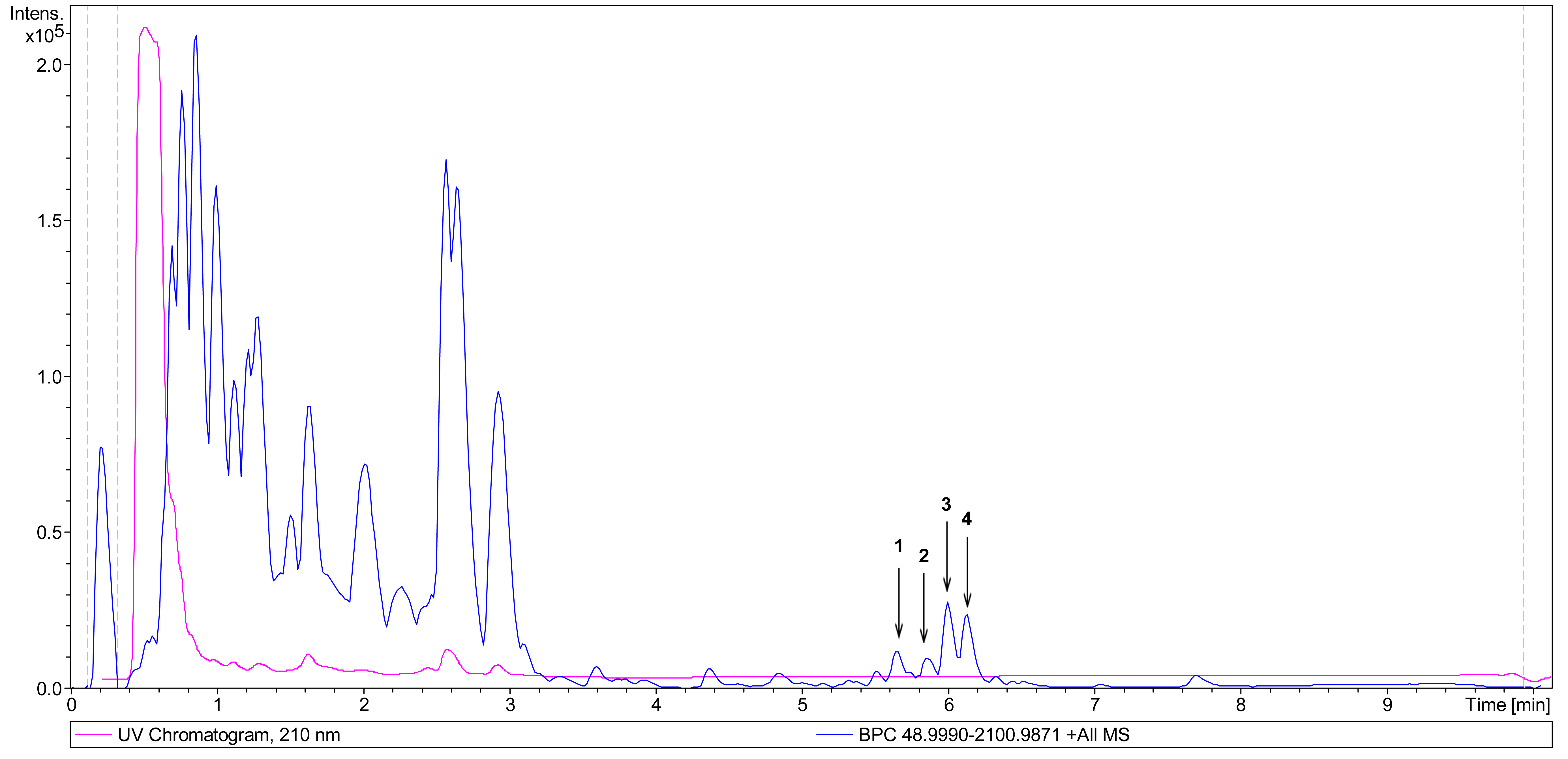

Supplement: Fig S5 — High-performance liquid chromatography–mass spectrometry profile of 225TR culture extracts. [file mbt20008-0716-sd5.tiff]

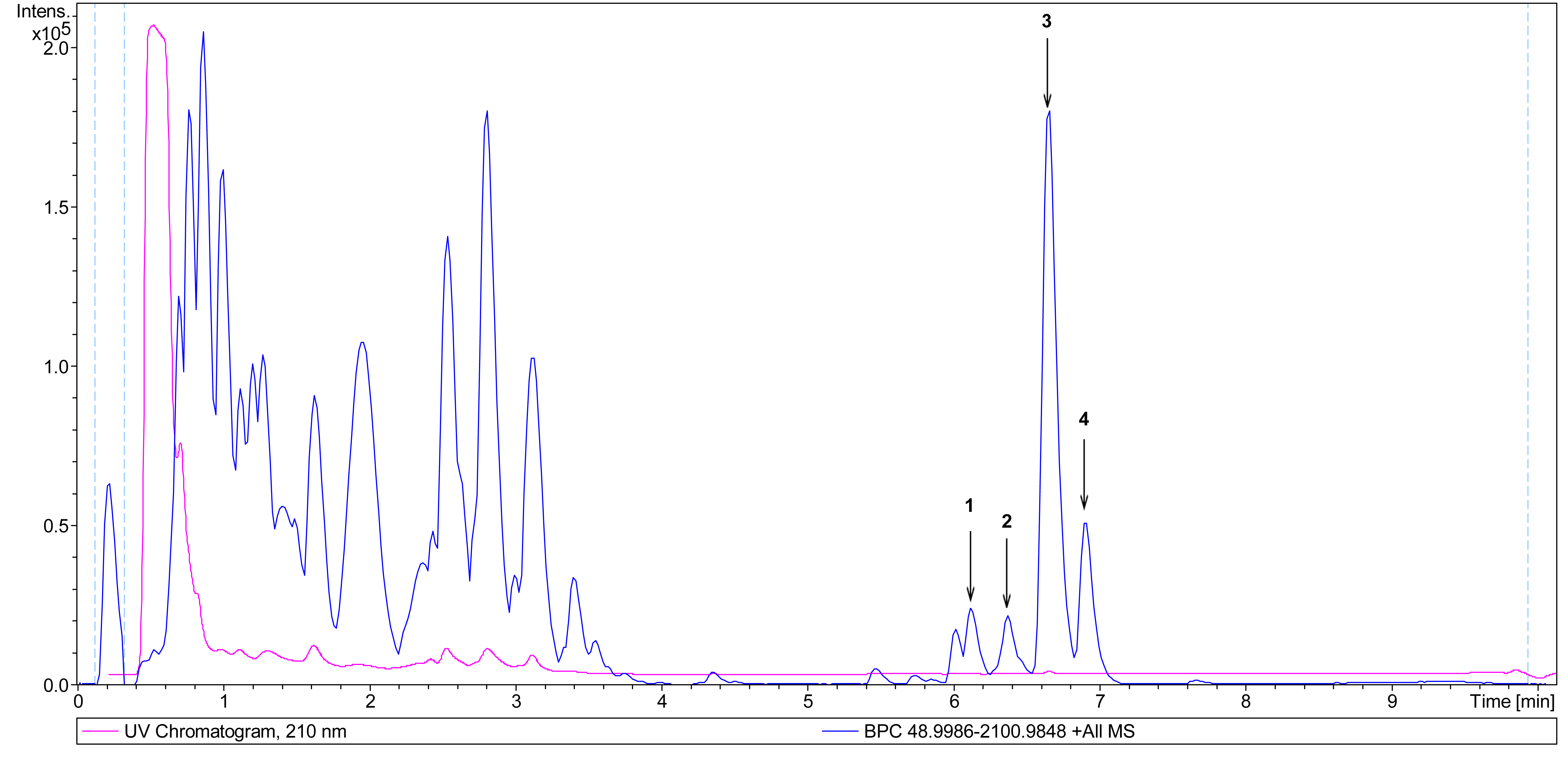

Supplement: Fig S6 — High-performance liquid chromatography–mass spectrometry profile of 250J culture extracts. [file mbt20008-0716-sd6.tiff]

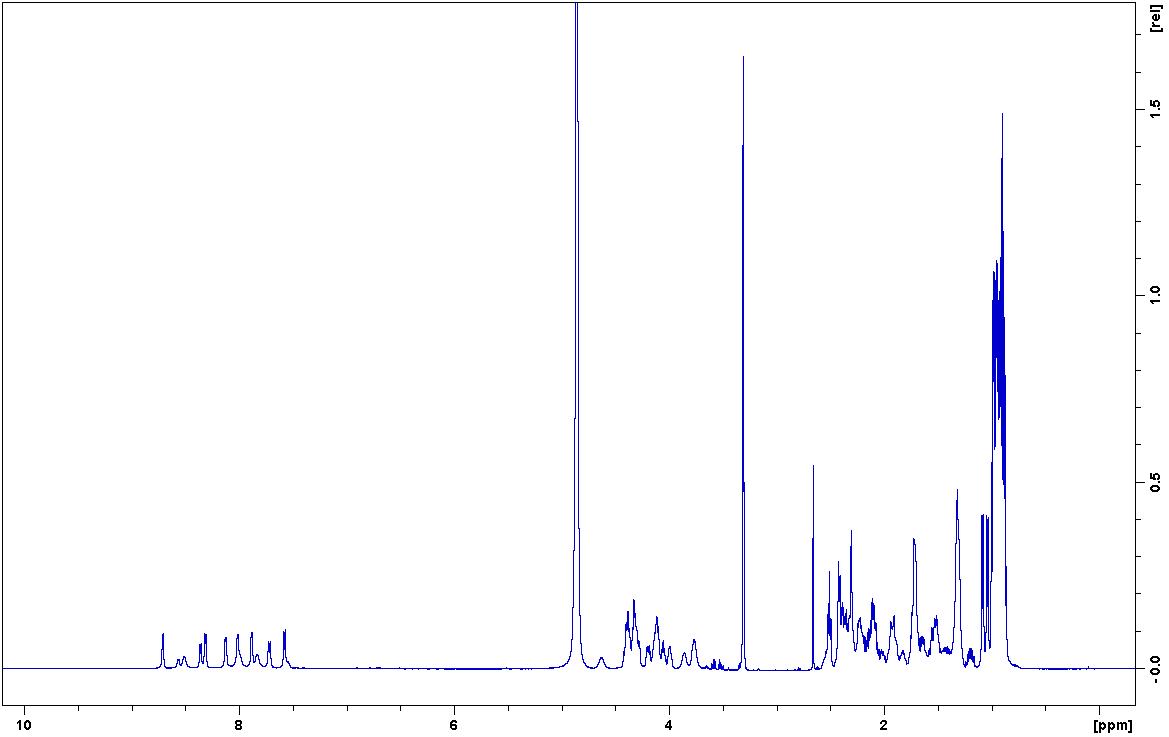

Supplement: Fig S7 — Nuclear Magnetic Resonance (NMR) of xantholysin A in MeOD. [file mbt20008-0716-sd7.jpg]

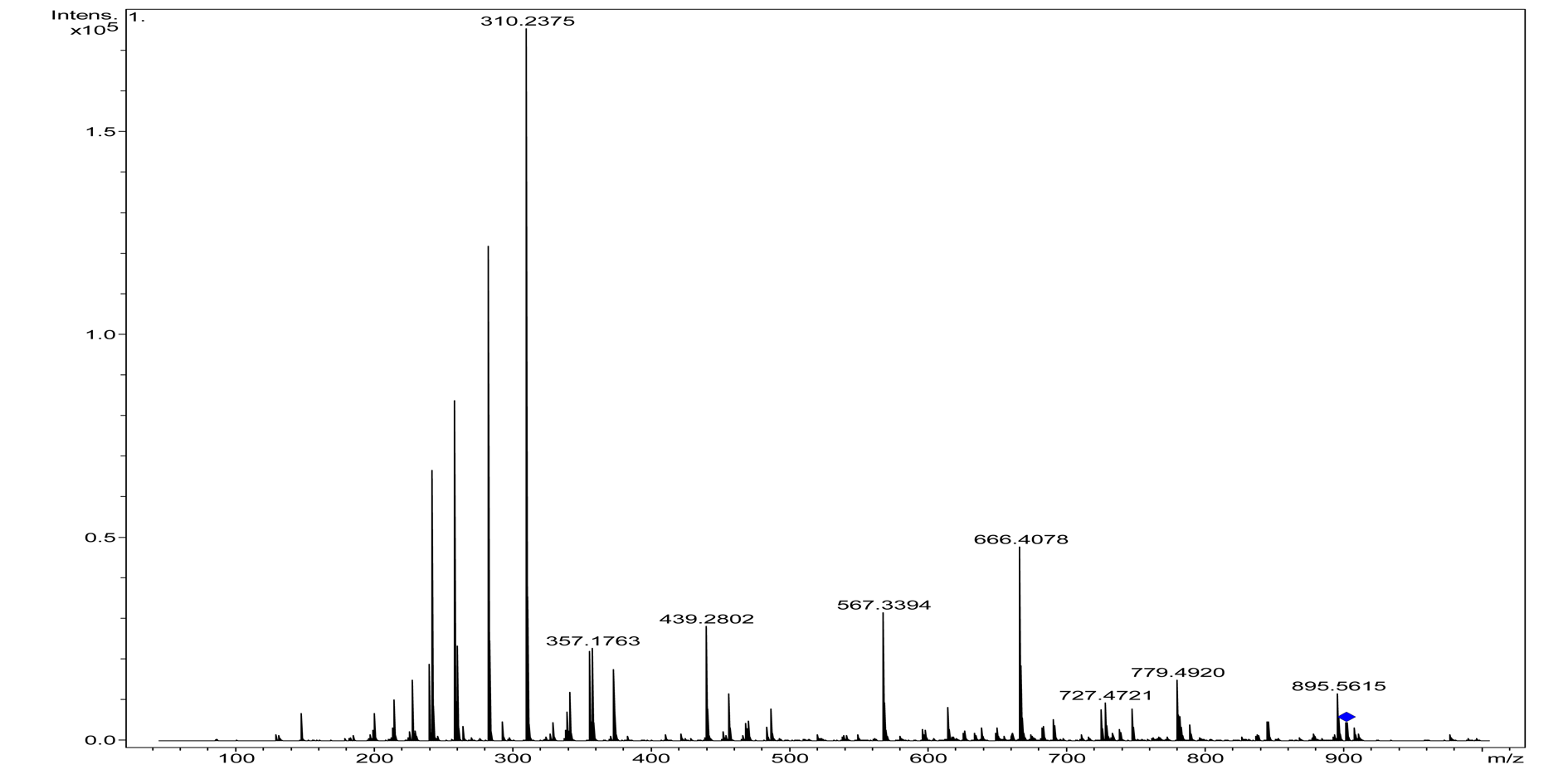

Supplement: Fig S8 — Tandem mass spectrometry analysis of xantholysin C. The analyses were performed to confirm the difference in the lipid tail between xantholysin A and xantholysin C, proposed by (Bernal et al., 2013). [file mbt20008-0716-sd8.tif]

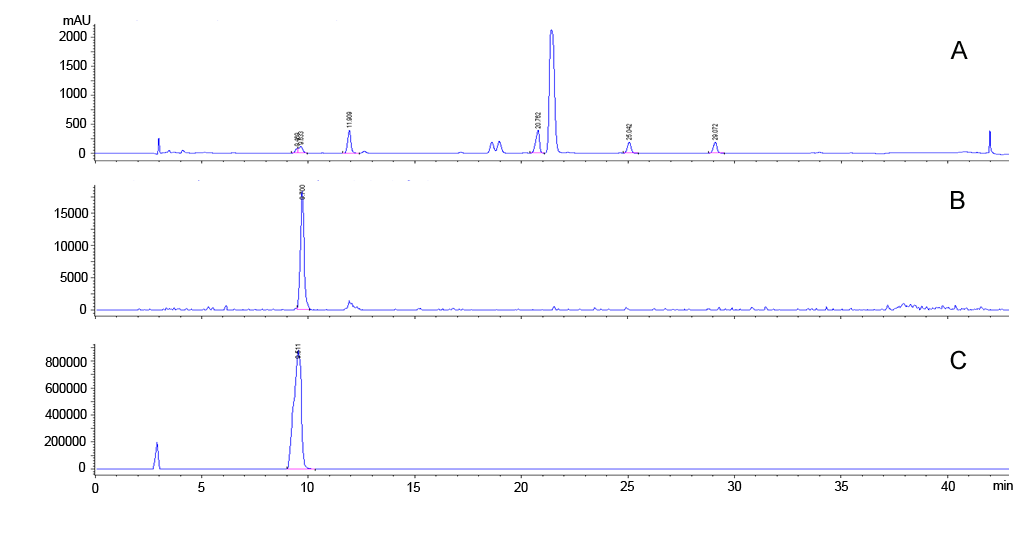

Supplement: Fig S9 — Marfey analysis of D-Ser, (A) xantholysin A MS, (B) positive ion of D-Ser in xantholysin A and (C) positive ion of D-Ser control. [file mbt20008-0716-sd9.tif]

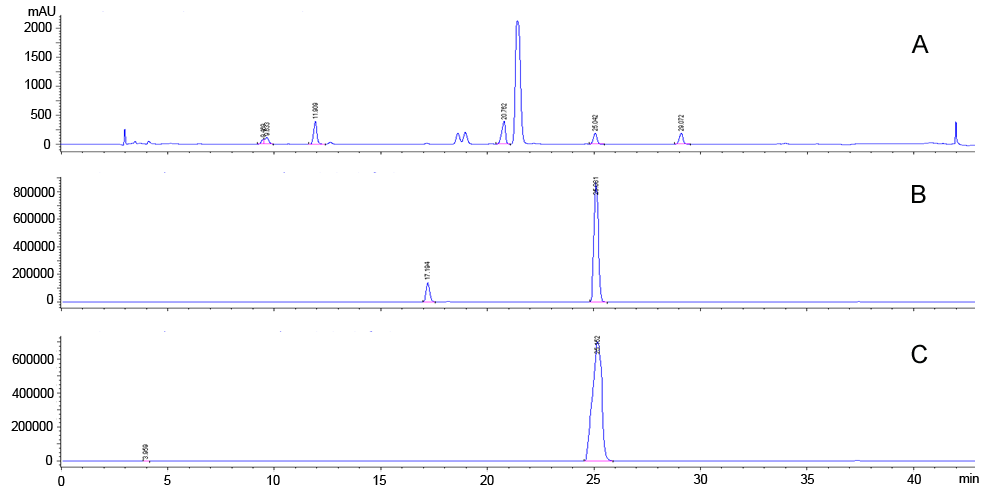

Supplement: Fig S10 — Marfey analysis of D-Val. (A) xantholysin A MS, (B) positive ion of D-Val in xantholysin A and (C) positive ion of D-Val control. [file mbt20008-0716-sd10.tif]

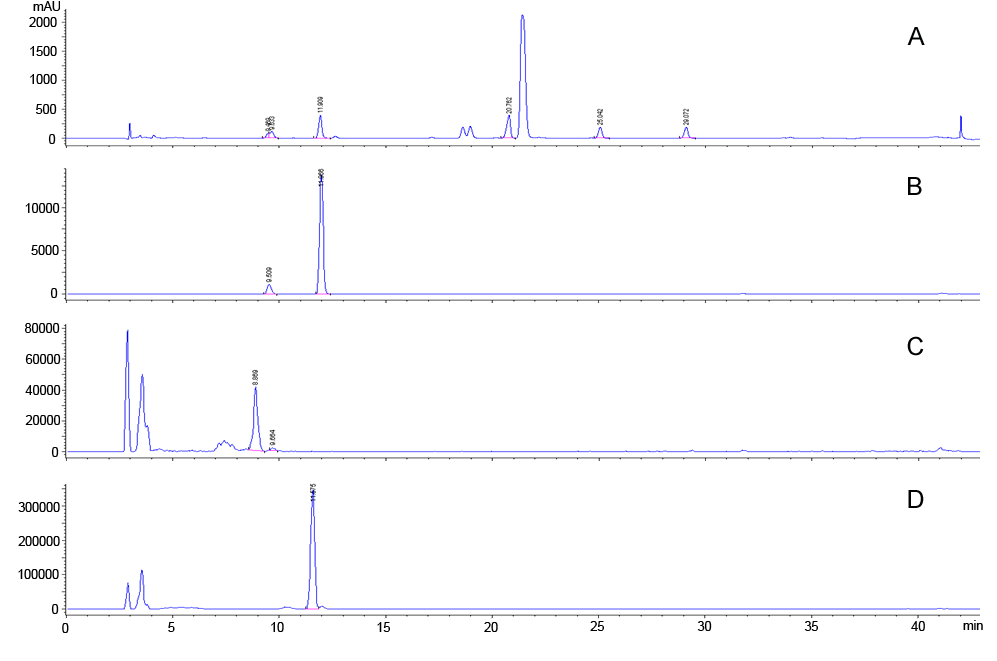

Supplement: Fig S11 — Marfey analysis of L- and D-Glu/Gln. (A) xantholysin A MS, (B) positive ion of D- and L-Glu/Gln in xantholysin A, (C) positive ion of D-Glu/Gln control and (D) positive ion of L-Glu/Gln control. [file mbt20008-0716-sd11.tif]

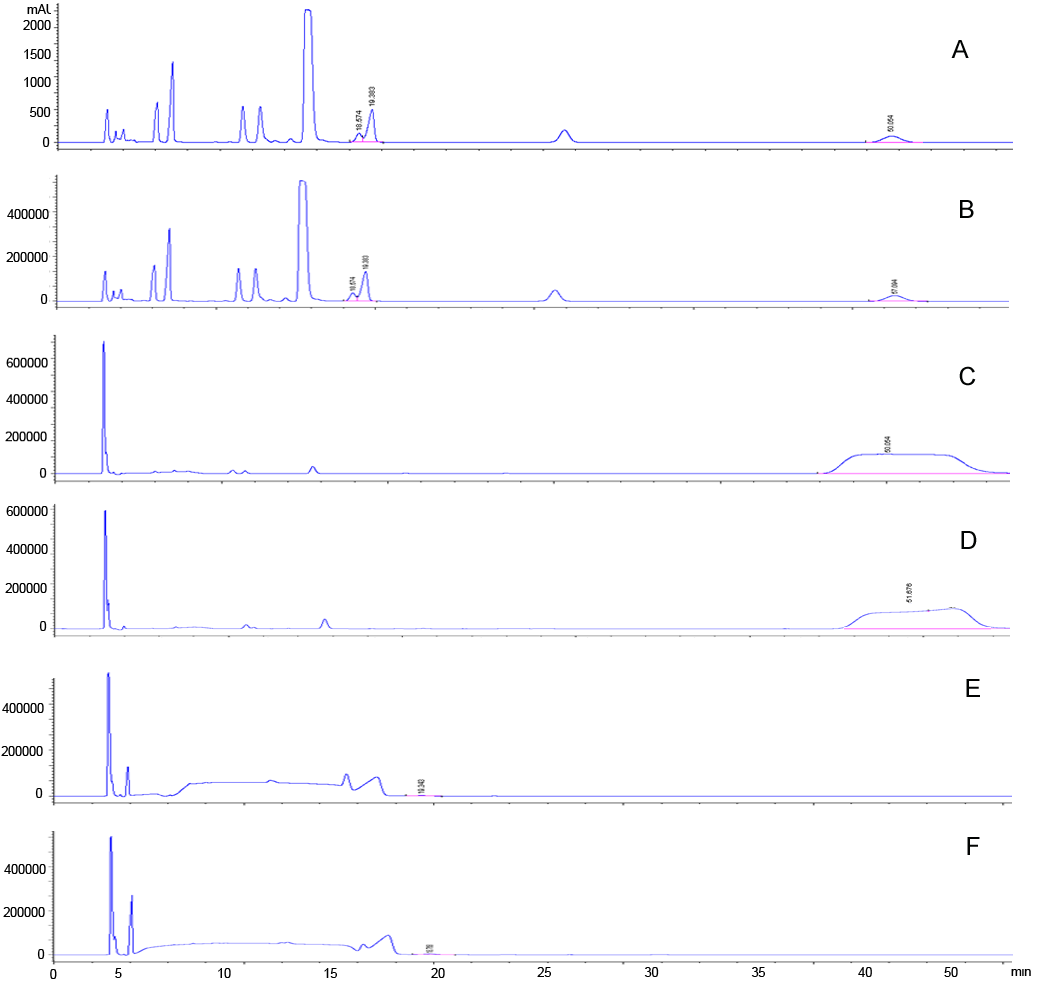

Supplement: Fig S12 — Marfey analysis of D- and L-Ile/Leu. (A) xantholysin A MS, (B) positive ion of D- and L-Leu/Ile in xantholysin A, (C) positive ion of D-Leu control, (D) positive ion of D-Ile control, (E) positive ion of L-Leu control and (F) positive ion of L-Ile control. [file mbt20008-0716-sd12.tiff]

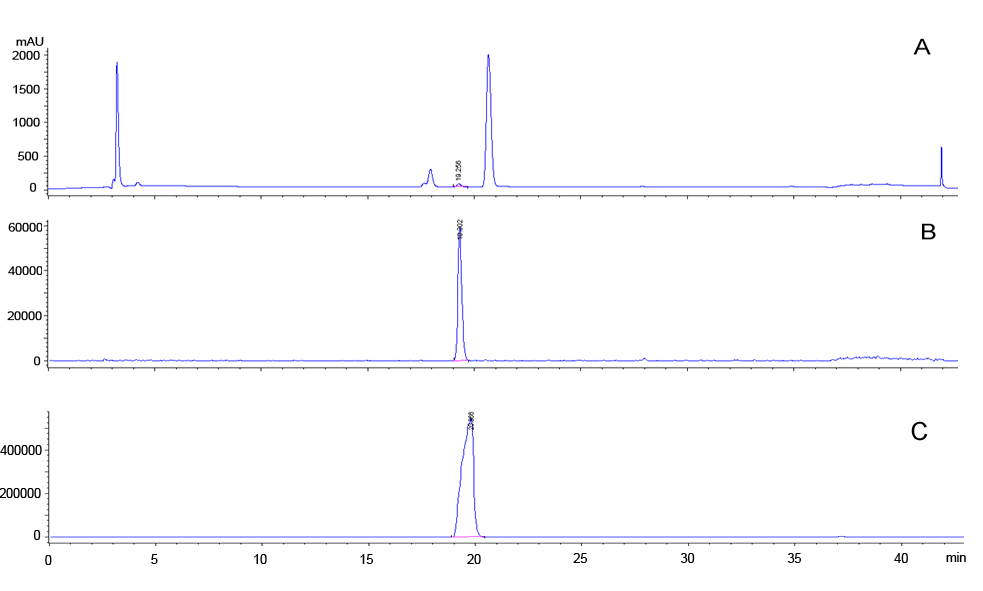

Supplement: Fig S13 — Marfey analysis of a fragment of 301 Da from partial hydrolysis. (A) fragment of 301 Da MS, (B) positive ion of L-Leu in fragment of 301 Da, (C) positive ion of L-Leu control. [file mbt20008-0716-sd13.tiff]

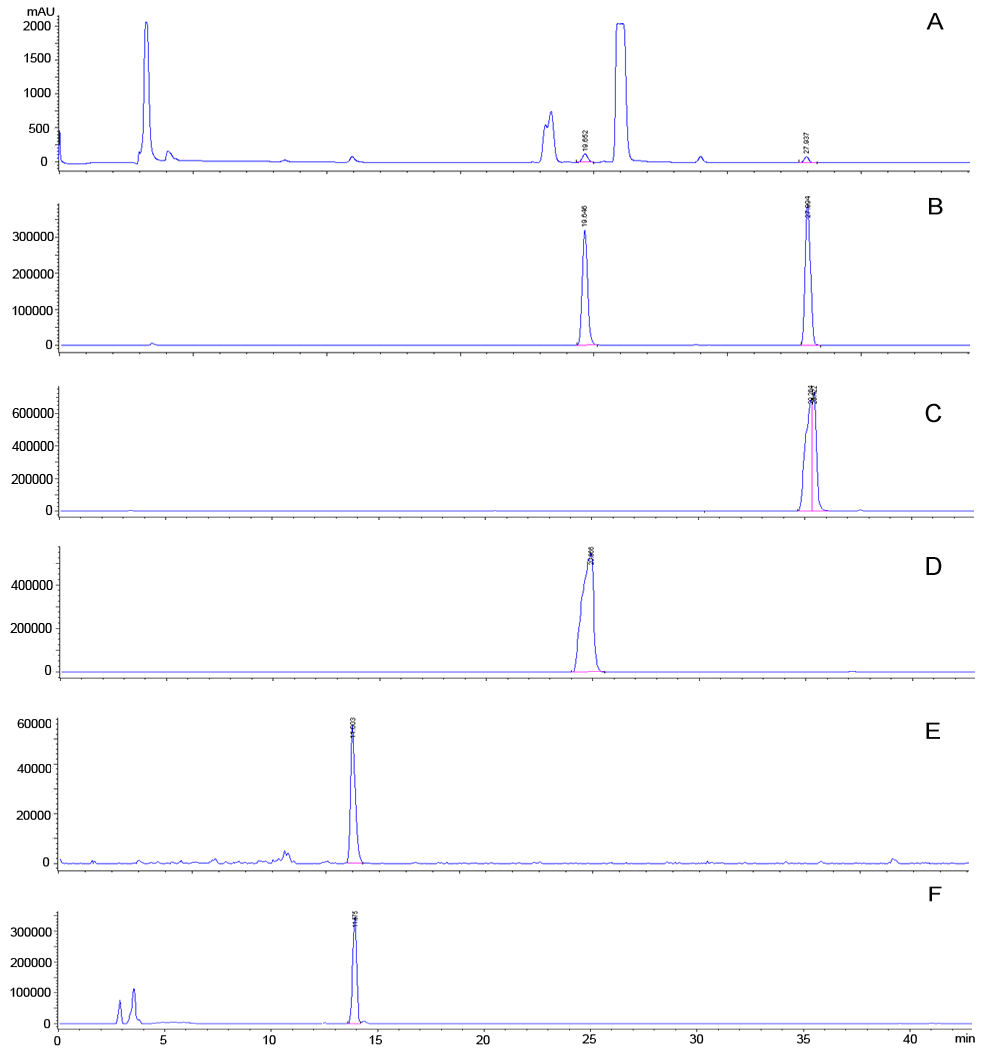

Supplement: Fig S14 — Marfey analysis of a fragment of 900 Da from partial hydrolysis. (A) Fragment of 900 Da MS, (B) positive ion of D-Leu and L-Leu in fragment of 900 Da, (C) positive ion of D-Leu control, (D) positive ion of L-Leu control, (E) positive ion of D-Glu in fragment of 900 Da, (F) positive ion of D-Glu control. [file mbt20008-0716-sd14.tiff]
