# Supplementary material for: Efflux pump-deficient mutants as a platform to search for microbes that produce antibiotics
Source: Microb Biotechnol. 2015 Jun 8;8(4):716–25. doi: 10.1111/1751-7915.12295 (PMC4476826; doi:10.1111/1751-7915.12295)
Supplement: Table S1 — Isolation of species capable of inhibiting DOT-T1E-18 growth. [file mbt20008-0716-sd15.docx]

Suppl. Table. Isolated species capable to inhibit DOT-T1E-18 growth

| Genus | Isolate | Closest type strain | Similarity | Isolation place |
| --- | --- | --- | --- | --- |
| *Pseudomonas* | 260O | *Pseudomonas sp.* | 100% | Olive soil |
|  | 215TR | *Pseudomonas nitroreducens* | 99% | Tinto River |
|  | 165W | *Pseudomonas alcaligenes* | 100% | Waste water treatment plant |
|  | 179W | *Pseudomonas pseudoalcaligenes* | 99% | Waste water treatment plant |
|  | 178W | *Pseudomonas aeruginosa* | 100% | Waste water treatment plant |
|  | 188W | *Pseudomonas stutzeri* | 100% | Waste water treatment plant |
|  | 216O | *Pseudomonas mendocina* | 99% | Olive soil |
|  | 250J | *Pseudomonas sp. strain 250J* | 100% | EEZ Garden |
|  | 254J | *Pseudomonas pecoglossicida* | 99% | EEZ Garden |
|  | 231PS | *P. putida* | 99% | Punta del Sebo |
|  | 217J | *Pseudomonas cuatrocienegasensis* | 99% | EEZ Garden |
|  | 265PS | *Pseudomonas monteilli* | 99% | Punta del Sebo |
| *Bacillus* |  |  |  |  |
|  | 146TR | *Bacillus sp.* | 99% | Tinto River |
|  | 278TR | *Bacillus pumilus* | 99% | Tinto River |
|  | 263TR | *Bacillus cereus* | 99% | Tinto River |
|  | 195TR | *Bacillus megaterium* | 99% | Tinto River |
|  | 270TR | *Bacillus thuringiensis* | 99% | Tinto River |
|  | 279MT | *Bacillus subtilis* | 99% | Muelle del Tinto |
|  | 176W | *Bacillus amyloquefaciens* | 99% | Waste water treatment plant |
|  | 187W | *Bacillus safensis* | 99% | Waste water treatment plant |
| *Lysinibacillus* |  |  |  |  |
|  | 234PS | *Lysinibacillus sp.* | 99% | Punta del Sebo |
|  | 249MT | *Lysinibacillus fusiformis* | 99% | Muelle del Tinto |
| *Shewanella* |  |  |  |  |
|  | 240TR | *Shewanella sp.* | 99% | Tinto River |
|  | 241PS | *Shewanella algae* | 99% | Punta del Sebo |
| *Alishewanella* |  |  |  |  |
|  | 255W | *Alishewanella sp.* | 98% | Waste water treatment plant |
| *Vibrio* |  |  |  |  |
|  | 225TR | *Vibrio proteoliticus* | 99% | Tinto River |
| *Acinetobacter* |  |  |  |  |
|  | 230MT | *Acinetobacter radioresistens* | 100% | Muelle del Tinto |
| *Rhodococcus* |  |  |  |  |
|  | 144TR | *Rhodococcus erythropyla* | 99% | Tinto River |
| *Alcaligenes* |  |  |  |  |
|  | 173W | *Alcaligenes faecalis* | 99% | Waste water treatment plant |
| *Aeromonas* |  |  |  |  |
|  | 182J | *Aeromonas hydrophila* | 99% | EEZ Garden |
|  | 167O | *Aeromonas media* | 99% | Olive soil |
| *Cupravidus* |  |  |  |  |
|  | 236MT | *Cupravidus sp.* | 96% | Muelle del Tinto |
| *Raoultella* |  |  |  |  |
|  | 208W | *Raoultella planticola* | 99% | Waste water treatment plant |
| *Enterobacter* |  |  |  |  |
|  | 207TR | *Enterobacter sp.* | 99% | Tinto River |
|  | 212J | *Enterobacter aerogenes* | 97% | EEZ Garden |
