# Supplementary material for: Efflux pump-deficient mutants as a platform to search for microbes that produce antibiotics
Source: Microb Biotechnol. 2015 Jun 8;8(4):716–25. doi: 10.1111/1751-7915.12295 (PMC4476826; doi:10.1111/1751-7915.12295)
Supplement: Table S2 — Antimicrobial compounds found in the 255W culture extracts. Positions correspond with the numbers that appear in Fig. S2. [file mbt20008-0716-sd16.docx]

Suppl. Table 2. Antimicrobial compounds found in the 255W culture extracts. Positions correspond with the numbers that appear in Suppl. Figure 3.

| **Position** | **Compound** | **Molecular Formula** | **Molecular Weight (g/mol)** |
| --- | --- | --- | --- |
| 1 | Plipastatin A1 | C_72_H_110_N_12_O_20_ | 1463.94 |
| 2 | Plipastatin A2 | C_73_H_112_N_12_O_20_ | 1477.73 |
| 3 | Plipastatin B1 | C_74_H_114_N_12_O_20_ | 1491.76 |
| 4 | Plipastatin B2 | C_75_H_116_N_12_O_20_ | 1505.79 |
| 5 | 4-L-Alaninesurfactin C1 | C_51_H_89_N_7_O_13_ | 1008.29 |
| 6 | 7-L-Valinesurfactin C1 | C_52_H_91_N_7_O_13_ | 1022.31 |
| 7 | Surfactin A o C1 | C_53_H_93_N_7_O_13_ | 1036.34 |
