# Supplementary material for: Efflux pump-deficient mutants as a platform to search for microbes that produce antibiotics
Source: Microb Biotechnol. 2015 Jun 8;8(4):716–25. doi: 10.1111/1751-7915.12295 (PMC4476826; doi:10.1111/1751-7915.12295)
Supplement: Table S3 — Antimicrobial compounds found in the 249MT extract. Positions correspond with the numbers that appear in Fig. S3. NPB, not produced by bacteria. [file mbt20008-0716-sd17.docx]

Suppl. Table 3. Antimicrobial compounds found in the 249MT extract. Positions correspond with the numbers that appear in Suppl. Figure 4. NPB – not produced by bacteria.

| **Position** | **Compound** | **Molecular Formula** | **Molecular Weight (g/mol)** |
| --- | --- | --- | --- |
| 1 | NPB (erythrolic acid D) | C_19_H_24_O_5_ | 332.39 |
| 2 | NPB | C_18_H_22_O_4_ | 302.36 |
| 3 | NPB | C_16_H_16_O_4_ | 272.29 |
| 4 | NPB | C_18_H_18_O_4_ | 298.33 |
| 5 | NPB | C_19_H_22_O_3_ | 298.36 |
