# Supplementary material for: Efflux pump-deficient mutants as a platform to search for microbes that produce antibiotics
Source: Microb Biotechnol. 2015 Jun 8;8(4):716–25. doi: 10.1111/1751-7915.12295 (PMC4476826; doi:10.1111/1751-7915.12295)
Supplement: Table S4 — Antimicrobial compounds found in the 225TR extract. Positions correspond with the numbers that appear in Fig. S4. DNP, not described in database of natural products. [file mbt20008-0716-sd18.docx]

Suppl. Table 4. Antimicrobial compounds found in the 225TR extract. Positions correspond with the numbers that appear in Suppl. Figure 5. DNP – not described in database of natural products.

| **Position** | **Compound** | **Molecular Formula** | **Molecular Weight (g/mol)** |
| --- | --- | --- | --- |
| 1 | Serratomolide C | C_28_H_50_N_2_O_8_ | 542.70 |
| 2 | 4-deoxy thiomarinol H | C_30_H_50_N_2_O_8_ | 566.72 |
| 3 | No DNP | C_30_H_52_N_2_O_8_ | 568.74 |
| 4 | No DNP | C_30_H_54_N_2_O_8_ | 570.75 |
