# Supplementary material for: Efflux pump-deficient mutants as a platform to search for microbes that produce antibiotics
Source: Microb Biotechnol. 2015 Jun 8;8(4):716–25. doi: 10.1111/1751-7915.12295 (PMC4476826; doi:10.1111/1751-7915.12295)
Supplement: Table S5 — Antimicrobial compounds found in the 250J extract. Positions correspond with the numbers that appear in Fig. 1. [file mbt20008-0716-sd19.docx]

Suppl. Table 5. Antimicrobial compounds found in the 250J extract. Positions correspond with the numbers that appear in Figure 1.

| **Position** | **Compound** | **Molecular Formula** | **Molecular Weight (g/mol)** |
| --- | --- | --- | --- |
| 1 | Xantholysin D | C_84_H_146_N_18_O_23_ | 1775.09 |
| 2 | Xantholysin B | C_83_H_144_N_18_O_23_ | 1761.04 |
| 3 | Xantholysin A | C_84_H_146_N_18_O_23_ | 1775.08 |
| 4 | Xantholysin C | C_86_H_148_N_18_O_23_ | 1802.0 |
