# Supplementary material for: Efflux pump-deficient mutants as a platform to search for microbes that produce antibiotics
Source: Microb Biotechnol. 2015 Jun 8;8(4):716–25. doi: 10.1111/1751-7915.12295 (PMC4476826; doi:10.1111/1751-7915.12295)
Supplement: Appendix S1 — Marfey analysis of xantholysin A. [file mbt20008-0716-sd20.docx]

**Suppl. Text 1**

Pseudomonas sp. strain 250J was fermented for 3 days in KIDO medium. One liter of the ferment was extracted with MEK and this extract was purified by repeated semi-preparative HPLC. Four compounds of the, until the date this work was performed, xantholysin family were detected: 1775.08 Da (xantholysin A), 1761.07 Da (xantholysin B), 1802.0 Da (xantholysin C), and 1775.09 Da (xantholysin D). Xantholysin A (C_84_H_146_N_18_O_23_) and xantholysin C (C_86_H_148_N_18_O_23_) were successfully isolated in sufficient quantity (16 mg and 5 mg respectively) to allow adequate NMR and MS-MS analysis (Suppl. Figures 6 and 7 NMR y MS-MS). The results confirmed that these compounds were xantholysin A and xantholysin C ([Li](#_ENREF_38) *[et al.,](#_ENREF_38)* [2013](#_ENREF_38)), with the single difference being the lipid tail moiety consisting of 3-hydroxydodec-5-enoate in xantholysin C rather than 3-hydroxydecanoate in xantholysin A — this difference accounts for the 26 Da mass increase in xantholysin C ([Li](#_ENREF_38) *[et al.,](#_ENREF_38)* [2013](#_ENREF_38)).

We decided to deeper study the configuration of the amino acid residues in xantholysin A by Marfey’s methodology. For that, we performed total and partial hydrolysis of the sample with HCl 6 N and 0.5 N, respectively (110 ºC, overnight), and subsequently derivatization of the hydrolysates with 1-fluoro-2,4-dinitrophenyl-5-l-valinamide (l-FDVA). This reagent reacts stoichiometrically with the primary amides in L- or D-amino acids, resulting in derivatives with different polarity which allowed us to identify most of the L- and D-amino acid configurations on the basis of comparison of their retention times with those of derivatized standards. Marfey analysis (Suppl. Figure 8-11) allowed us to identify by HPLC-UV-MS that the serine and the two valine residues were D-amino acids. The analysis also permitted us to identify that Isoleucine has an L-Ile configuration. In the case of Glu-Gln and Leu residues, we found ratios of L- and D- amino acids of 1:4 and 3:2, respectively. Partial hydrolysis and purification of the fragments identified the position of two D-Gln and one D-Glu and also one L-Leu and one D-Leu (Suppl. Figure 12-13), although this analysis was not sufficient to determine the complete structure of xantholysin A.

In an attempt to resolve the complete structure, bioinformatics analysis performed with NaPDoS ([Ziemert](#_ENREF_39) *[et al.,](#_ENREF_39)* [2012](#_ENREF_39)) using 250J sequenced genome ([Molina-Santiago](#_ENREF_40) *[et al.,](#_ENREF_40)* [2014](#_ENREF_40)) suggested that domains C12 and C13 (belonging to amino acids Leu and Gln) are LCL-C domains, which catalyze peptide bond formation between two L-amino acids. This analysis supported that these Leu and Gln residues are likely L-amino acids. The *in silico* analysis revealed the potential configuration of the last Gln residue and of the two Leu residues. Figure 1 shows the potential elucidation structure of xantholysin A although the L- or D- configurations of Leu9 and Leu11 are unknown at present.
